# With a Little Help From Their Friends: Endolithic Microbial Communities Modulate Mussel Survival, Behaviour and Molecular Responses Under Thermal Stress

**DOI:** 10.1111/mec.70524

**Published:** 2026-08-21

**Authors:** Sarah Bollina, Gerardo I. Zardi, Virginie Cuvillier, Chloé Tilliette, Emma Brigant, Anne‐Catherine Holl, Katy R. Nicastro

**Affiliations:** ^1^ LOG—Laboratoire d'Océanologie et de Géosciences, IRD, UMR 8187, CNRS, Univ. Littoral Côte d'Opale Univ. Lille Lille France; ^2^ Department of Zoology and Entomology Rhodes University Grahamstown South Africa; ^3^ CNRS, UMR 8198 Univ. Lille Lille France

**Keywords:** extreme events, gene expression, heat stress, HSP, immune detection, marine

## Abstract

As thermal stress intensifies under global climate change, ecosystems worldwide are undergoing profound transformations that disrupt biological processes, species distributions and biodiversity, with coastal marine systems particularly vulnerable. Organisms have evolved behavioural, physiological and morphological strategies to cope with extreme conditions, while species interactions, especially symbioses, can further shape responses to stress. These symbioses often shift along a continuum from mutualism to parasitism depending on environmental context. One notable example is the dynamic relationship between bivalves and their endolithic microbial communities. Here, we investigated how variation in visible endolithic shell corrosion relates to differences in mussel survival, behaviour and molecular responses during repeated aerial heat stress. Mussels with endolithic shell corrosion consistently showed enhanced survival. Behaviourally, heat stress impaired mobility (i.e., net and gross distance travelled) and byssal thread production (i.e., number of byssal threads), but mussels with endoliths exhibited comparatively buffered responses to these effects. At the molecular level, mussels with endoliths showed lower induction of HSP24 and HSP70 after thermal stress, but higher baseline HSP60 and HSP90 expression, suggesting distinct stress‐response pathways. Together, our findings reveal consistent, multi‐level differences in host responses associated with endolithic shell condition across behavioural and molecular traits and survival, suggesting that endoliths may contribute to enhanced thermal resilience and, consequently, could play a role in shaping the resilience of mussel populations under future climate‐driven heat stress. More generally, these results highlight the critical role of interspecific interactions, including host‐associated microbial assemblages, in shaping adaptive responses in hosts.

## 1 Introduction

As thermal stress intensifies with climate change, coastal marine ecosystems are undergoing profound and sometimes irreversible shifts, altering biological processes, ecosystem functioning, species distributions and biodiversity (Ainsworth et al. 2020; Urban 2024; Wernberg et al. 2025). Intertidal shores are among the most thermally variable and stressful habitats (Helmuth and Hofmann 2001; Stillman et al. 2025; Wernberg et al. 2024), and organisms inhabiting these coastal environments have evolved diverse strategies and complex solutions to cope with environmental extremes, including behavioural, physiological and morphological adaptations (Dong 2023; Marshall et al. 2011; Ng et al. 2021; Stillman et al. 2025).

Beyond individual‐level traits, species interactions—particularly symbiotic relationships—can play a crucial role in modulating host responses to environmental stressors, enabling adaptations and survival in stressful conditions (Buchner 1953; Ezenwa et al. 2012; Feldhaar 2011; Thiel and Baeza 2001; Wong et al. 2015). While symbioses are traditionally categorised as mutualistic, commensal or parasitic (Daskin and Alford 2012; Hosokawa and Fukatsu 2020), many symbioses shift along a continuum of interaction depending on the situation (Daskin and Alford 2012). In these context‐dependent symbioses, the effect on the host varies with environmental conditions, where the symbionts might be beneficial under some conditions but harmful under others (Daskin and Alford 2012; Fellous and Salvaudon 2009). A compelling example is the relationship between intertidal bivalves and endolithic microbial communities (Collins et al. 2023; Ćurin et al. 2014; Kaehler 1999; Mikac et al. 2021). These widespread endolithic communities corrode the shell of coastal bivalves (e.g., Kaehler and McQuaid 1999), reducing survival and compelling mussels to make critical energy trade‐offs between shell repair, reproduction, growth and attachment strength (Zardi et al. 2009). Yet under heat stress, shell‐corroding endoliths can confer advantages through shell whitening, thereby increasing the albedo and reducing heat absorption and internal body temperature (Gehman and Harley 2019; Monsinjon et al. 2026; Zardi et al. 2016) and ultimately increasing survival during heat waves (Zardi et al. 2024). Here, we investigate whether the influence of these endolithic communities extends beyond morphological alterations such as shell whitening to also modulate the molecular and behavioural responses of their hosts.

At the molecular level, one of the most conserved and widespread mechanisms throughout the animal kingdom for coping with heat stress is the induction of the heat shock response—a cellular pathway that rapidly upregulates protective proteins to maintain cell functions under extreme conditions (Santoro 2000; Shan et al. 2020; Singh et al. 2024). Heat shock proteins (HSPs) act as molecular chaperones that prevent protein denaturation, refold damaged proteins and maintain enzymatic activity, thereby safeguarding key metabolic pathways during heat exposure (Ioannou et al. 2009; Shan et al. 2020; Singh et al. 2024). Across taxa—from invertebrates and fish to mammals—the upregulation of HSPs during thermal stress is both a defining feature of acclimation and a reliable indicator of organismal resilience under prolonged or repeated heat challenges, highlighting its ecological and evolutionary significance. The upregulation of HSPs is one of the most widely described heat acclimation mechanisms in metazoans (Feder and Hofmann 1999; Sørensen et al. 2003). Recent work in bivalves has further shown that heat stress induces coordinated molecular stress responses involving HSPs alongside apoptosis, and tissue‐specific transcriptional reprogramming, highlighting the integrated nature of thermal stress responses (Bonesteve et al. 2025; Li et al. 2025; Yao et al. 2025).

HSP induction is often not solely a response to thermal stress. HSP60 and HSP70 notably play important roles in innate immune defence, their upregulation being frequently triggered by pathogen exposure, infection or immune stimulation (Bolhassani and Agi 2019; Kaufmann et al. 1991; Zhang and Qi 2025; Zhou et al. 2010). Consistent with this, transcriptomic studies in marine bivalves have shown that HSP60 and HSP70 are frequently co‐regulated with immune and stress‐response pathways under thermal stress (Li et al. 2025; Matsusaka et al. 2026). Within symbiotic associations, immune detection of the microbial partner can therefore lead to elevated baseline HSP expression, thereby potentially enhancing tolerance or proactively buffering the host against the deleterious effects of elevated temperatures (Barman et al. 2023; Selbach et al. 2020). This aligns with the broader concept of cross‐tolerance, whereby exposure to one stressor increases resilience to another through shared molecular pathways (Barshis et al. 2013; Collins et al. 2021). Increasing evidence across taxa indicates that immune activation and thermal resilience are often mechanistically intertwined rather than independent processes. Elevated constitutive or inducible expression of HSPs can enhance tolerance to subsequent heat stress through molecular priming or transcriptional frontloading of stress‐response pathways (Barshis et al. 2013; Collins et al. 2021; Greve et al. 2026). Such pre‐activation of cellular stress responses has been associated with improved physiological performance under acute or combined stressors, where shared stress‐response pathways confer protection against multiple environmental challenges (Barrett et al. 2022; Sun et al. 2025).

Inducible HSPs are primarily expressed under stress and serve as reliable biomarkers of protein damage and adverse environmental conditions (Feder and Hofmann 1999; Lindquist 1986). In marine invertebrates, their expression is widely used as a biomarker (Fabbri et al. 2008), with gene families such as HSP24, HSP60, HSP70 and HSP90 frequently being upregulated in response to diverse stressors (Anestis et al. 2007; Fabbri et al. 2008; Lockwood et al. 2010; Nielsen et al. 2021; Payton et al. 2016; Snyder et al. 2001). These gene families differ in their induction dynamics and stress specificity. Small HSPs such as HSP24 respond rapidly and are sensitive to heat stress, serving as early indicators of acute thermal challenge (Lockwood et al. 2010). HSP70 is also strongly heat‐inducible, but its expression typically peaks later, reflecting sustained defence against prolonged heat exposure (Feidantsis et al. 2020; Nielsen et al. 2021; Xu et al. 2014, 2022). Beyond its chaperone function, HSP70 can also be secreted by cells and play a key role in innate immunity, binding to Toll‐like receptor, expressed by immune cells and activating downstream immune responses (Junprung et al. 2024; Zhang and Qi 2025). By contrast, HSP60 and HSP90 are less heat‐specific, instead contributing to broader responses to diverse and often chronic stressors, with induction usually occurring in later stages of the stress response (Ding et al. 2018; Feidantsis et al. 2020; Georgoulis et al. 2025; Ivanina et al. 2016; Kim et al. 2009; Liu et al. 2019; Liu et al. 2016; Shi et al. 2016; Xu et al. 2014, 2022). Taken together, these four HSPs provide a multifaceted perspective on stress physiology, capturing both rapid, heat‐specific responses and more general, long‐term reactions to persistent stress.

In addition to molecular mechanisms, behavioural responses provide another crucial layer of protection against thermal stress (Chapperon and Seuront 2012; Ng et al. 2021; Virgin et al. 2025). Such behavioural adjustments are often rapid and reversible, providing a fast first line of defence, complementing slower‐acting physiological and molecular responses. In coastal systems, behaviours such as microhabitat selection, burrowing and shell orientation can significantly reduce exposure to extreme temperatures, highlighting the interplay between behavioural plasticity and cellular stress response in these dynamic environments (Beever et al. 2017). For instance, many intertidal species seek shade, burrow or alter their position to avoid overheating (Beever et al. 2017; Ng et al. 2021). Social behaviours such as aggregation can further buffer individuals from extreme conditions by creating localised microclimates with more favourable temperature or humidity (Chapperon and Seuront 2012; Ng et al. 2021; Nicastro et al. 2012).

Here, we examine how microbial endolithic communities influence mussel behaviour, survival and molecular responses to thermal stress during repeated periods of aerial heat exposure. Specifically, we tested the following hypotheses: (i) Mussels with endoliths show greater tolerance to heat stress, reflected in enhanced behavioural traits such as increased byssal thread production, higher motility and larger aggregate formation under thermal stress. (ii) Mussels with endoliths have higher survival under heat stress compared to those without. (iii) Mussels without endoliths exhibit low baseline HSP expression but show a strong increase in HSP expression following heat exposure. (iv) Mussels with endoliths exhibit elevated baseline HSP expression due to the presence of the microbial community, with minimal or no further increase following heat exposure.

## 2 Methods

### 2.1 Study Area and Model Species

**FIGURE 1:**
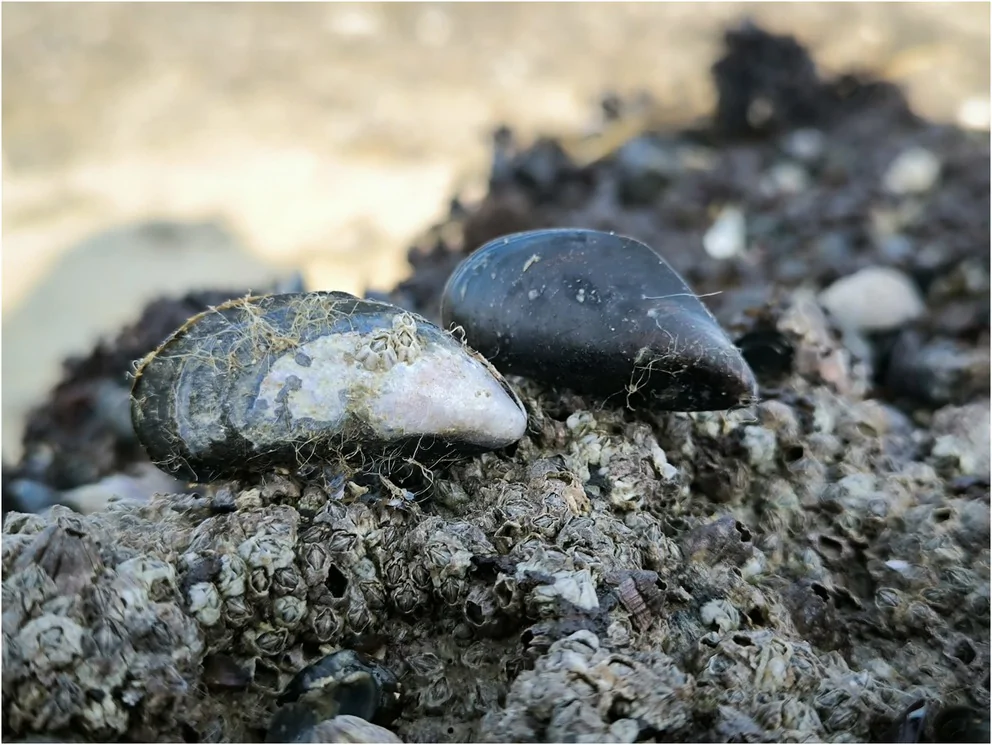
The blue mussel *Mytilus edulis* with (on the left) and without (on the right) endoliths.

Intertidal mussels ( *Mytilus edulis* ) were collected from Pointe aux Oies, Wimereux, France (50°47′12.3″ N 1°36′15.7″ E), where they naturally exhibit varying levels of shell corrosion through endolithic microbes. Endolithic shell corrosion is a commonly used indicator of endolithic activity in intertidal bivalves (e.g., Gehman and Harley 2019; Kaehler 1999; Kaehler and McQuaid 1999; Wyness et al. 2022, 2025), and shell corrosion can progress through five stages (Kaehler 1999): (A) a clean shell with intact periostracum and prominent growth lines; (B) the central area of the shell shows signs of erosion and the outer growth lines become less defined; (C) shells with signs of erosion spreading past the central parts, with grooves and pits on the surface; (D) heavily pitted shells that are becoming deformed, with almost completely absent outer growth lines; (E) extremely pitted shells, deformed and brittle, eventually developing visible holes. Here, mussels of stage A (non‐corroded) and stage D (corroded) were used (Figure 1). Prior to experiments, mussels were cleaned of epifauna and acclimated for 72 h in natural seawater at in situ conditions (*T* = 10°C, *S* = 33 PSU), gradually increased to 15°C. Air bubblers maintained oxygenation. Mussels were selected based on shell corrosion (high endolithic shell corrosion vs. no visible corrosion), and standardised for size (2.5–3 cm shell length).

### 2.2 Experimental Set‐Up

**FIGURE 2:**
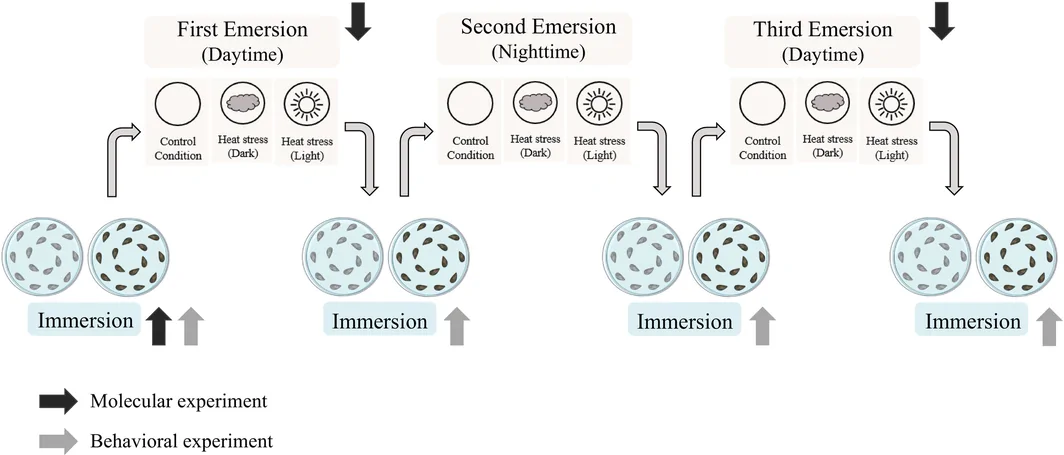
Experimental set‐up following the tidal cycles, with black arrows indicating time of sample collection for the molecular part and grey arrows indicating time of data collection for the behavioural part.

To simulate natural tidal cycles, mussels were subjected to alternating immersion and emersion phases (Figure 2), with stable aquatic conditions during simulated high tide (water temperature constantly at 15°C), while during low tide they were exposed to one of the three different thermal treatments, reflecting natural low tide events. The thermal treatments were either a hot sunny day (heat stress light: *T* = 33°C, with irradiance provided by artificial lamps and quantified as photosynthetic photon flux density [PPFD], ranging from 893 to 908 μmol photons m^−2^ s^−1^), a hot cloudy day (heat stress dark: *T* = 33°C, without irradiance) or a control treatment (control: *T* = 17°C, without irradiance), characterised by optimal temperatures for *M. edulis* (Bayne 1973; Incze et al. 1980).

For the first and third emersions (daytime), the hot sunny day was simulated using halogen lamps, heating the stones underneath from 20°C in the beginning to 33°C at the end of the emersion (Table S1), while the hot cloudy day and control were simulated using an incubator (MIR‐154‐PE) in dark mode. Importantly, temperature profiles for the hot sunny day were continuously recorded (Table S1), and these temperature dynamics were then used to replicate identical thermal exposure intervals and temperature increases in the hot cloudy treatment, ensuring comparable thermal stress regimes across light and dark conditions. The second emersion (nighttime) simulated a hot summer night, characterised by the absence of solar radiation, but maintained elevated temperatures. The respective temperatures were lowered (20°C for the heat treatments and 14°C for the control), reflecting average nocturnal temperatures during periods when daytime temperatures reached 33°C as well as typical spring conditions (mean values for the years 2021–2023 in Wimereux, InfosClimat (https://www.infoclimat.fr/)).

Across all experiments, equal numbers of mussels were used per treatment and condition, with replication balanced across weekly runs. Specifically, behavioural experiments used 84 individuals per condition per weekly run, while molecular and survival experiments each used 60 individuals per condition per weekly run. All experiments were repeated over three weekly runs, ensuring consistent replication across treatments and conditions.

### 2.3 Molecular Experiment

To mimic natural tidal rhythms, mussels were subjected to a cycle of 7 h of immersion followed by 5 h of emersion, repeated three consecutive times (Figure 2). This 12 h cycle was chosen to approximate semi‐diurnal tidal conditions experienced by mid‐intertidal *M. edulis* . The emersion duration was based on in situ assessments of mussel exposure conditions, where aerial exposure commonly lasts approximately 5–6 h depending on tidal coefficient and vertical position on the shore (Zardi et al. 2021). This experimental sequence was carried out on three separate occasions over a three‐week period, between 16 and 28 May 2024. Across the three experimental runs, each treatment and condition was represented by two independent replicates per run, resulting in a total of six replicates per treatment and condition over the course of the experiment.

Mussels were separated by condition (presence or absence of endoliths) and treatment (control, heat dark, heat light) and were strictly kept separated during the whole experiment. After the first high‐tide simulation (1st immersion; Figure 2), mussels from each condition and treatment were randomly collected and considered as the baseline time point, prior to any treatments. The individuals were immediately sexed, and only males were retained, as sex‐specific differences in heat‐shock protein expression have previously been reported in bivalves (Meistertzheim et al. 2009; Nicastro et al. 2023; Zhang et al. 2022). Restricting the analyses to males therefore reduced potential variability. Because expression patterns are most pronounced in mantle tissue (González‐Riopedre et al. 2007), mantle tissue was dissected from each male mussel and preserved in RNAlater, stored at 4°C. The same procedure (random collection and processing of male mantle tissues) was followed after the first and third daytime emersions. Molecular measurements were restricted to three biologically relevant timepoints: before heat exposure to represent the pre‐exposure state (baseline), after the first heat exposure (first emersion) and after repeated heat exposure following the third emersion (third emersion). By the end of the third cycle, six male individuals per time point and treatment group (*n* = 6) were obtained and subsequently processed.

RNA was extracted using NucleoSpin RNA Plus kits (Macherey Nagel), following the manufacturer's guidelines. Following the extraction and quality and quantity controls, cDNA was synthesised from 1 μg of total RNA, using the RevertAid RT Kit (ThermoScientific). The expression levels of target genes HSP24, HSP60, HSP70 and HSP90 were assessed using quantitative real‐time PCR (qPCR, LightCycler 480, Roche, using iTag Universal SYBR Green Supermix from Bio‐Rad) with gene‐specific primers, normalised against the expression of two reference genes (rpl14 and rps4, Salatiello et al. 2022; Table S2).

#### 2.3.1 Statistical Analysis

To test for the effects of treatment (control, heat dark, heat light), timepoint (baseline, first emersion (daytime), third emersion (daytime)) and condition (presence or absence of endoliths) on each heat shock protein expression, the Aligned Rank Transform (ART) method, a non‐parametric factorial analysis, implemented in the ARTool R package (Wobbrock et al. 2011) was used. This allows testing for main effects and interactions in a full factorial design, providing a robust nonparametric framework to assess complex interactions. To further explore how treatment and endolithic shell corrosion jointly influence HSP expression at each stage of heat exposure, the dataset was split by timepoint and a separate ART model for each timepoint was performed. Significant interactions or main effects were explored with a post hoc pairwise comparison, using the art.con() function, implemented in the ARTool R package. Multiple testing correction was applied using the Holm method.

All statistical analyses were conducted using R version 4.3.2.

### 2.4 Behavioural Experiment

To mimic natural tidal rhythms, mussels were subjected to a cycle of 6 h of immersion followed by 6 h of emersion, repeated three consecutive times (Figure 2). This experimental sequence was carried out three times, over a three‐week period (between 8th and 24th July 2024). Behavioural measurements could only be conducted while mussels were active (i.e., when underwater; Figure 2); therefore, all observations were performed during high‐tide simulations (immersion). During these immersions, mussels (*n* = 14) were placed in each behavioural glass arena (⌀ 26 cm) according to treatment (control, heat dark, heat light) and condition (presence or absence of endolithic shell corrosion). Two arenas per treatment and condition were run per week. At the end of the three‐weekly experiment, this resulted in a total of six replicated arenas per treatment and condition (*n* = 6). Each arena was filled with 1 L of seawater, previously acclimated to 15°C. All mussels were positioned equidistant, in concentric circles. During the high tide simulations, arenas were kept under static conditions and at constant water temperature (15°C).

Behavioural arenas were photographed every minute using GoPro Hero 10 cameras. Movement (gross and net distances crawled) was analysed from GoPro images using the TrackMate plugin in ImageJ (Tinevez 2017). Since shorter distances were considered oscillations around their centre, rather than active movement (Uguen et al. 2022), distance crawled was defined as any movement > 1 cm. At the end of each high tide simulation (i.e., after 6 h), the percentage of mussels within an aggregation, defined as a minimum of two individuals in physical contact (Nicastro et al. 2007), was assessed. As a proxy for attachment strength, the number of byssal threads produced by each individual was counted, including threads attached to the glass arena and to conspecifics. Each arena was measured at four timepoints, yielding repeated measures over time for the same experimental units.

#### 2.4.1 Statistical Analysis

For all behavioural traits (movement, aggregation, attachment), differences between weeks were assessed using a *t*‐test. As no significant differences were detected, data was pooled across weeks, resulting in a total of six replicated arenas per treatment and condition. Prior to statistical analyses, potential outliers were screened within each experimental condition, using *z*‐scores and individuals with *z*‐scores > 3 were considered outliers and removed before averaging (Iglewicz and Hoaglin 1993). For movement and byssal thread production, the mean value per behavioural arena was calculated (based on *n* = 14 mussels per arena), while aggregation was expressed as the percentage of aggregated mussels per arena (*n* = 6).

To assess the effect of treatment (control, heat dark, heat light), timepoint (control, first emersion (daytime), second emersion (nighttime), third emersion (daytime)) and condition (presence or absence of endolithic shell corrosion), a generalised linear mixed‐effects model (glmmTMB) was used (Brooks et al. 2017). A full factorial design was used, allowing comparisons across all treatment, timepoint and condition levels. Arena ID was set as a random intercept to account for the repeated measures of the same arenas over time. Differences between the treatment groups and conditions over time were evaluated using estimated marginal means (EMMs), using the emmeans package (Lenth 2016). Post hoc pairwise comparisons were conducted across all levels of the three‐way interaction, with adjustments for multiple testing using the Tukey method.

### 2.5 Survival Experiment

In a parallel experimental set‐up, mussels (*n* = 20 per treatment and condition) were used to assess survival following repeated heat exposure. Experimental treatment groups and cycles were identical to those previously described, with three repeated cycles of immersion and emersion over a three‐week period. However, no mortality was observed after three cycles, which led to the inclusion of an additional fourth low‐tide exposure under the same conditions. Survival rates were therefore assessed only after this additional fourth low‐tide heat exposure.

#### 2.5.1 Statistical Analysis

To assess the effect of treatment (control, heat dark, heat light), and condition (presence or absence of endolithic shell corrosion), a generalised linear model (GLM) was used, followed by pairwise comparisons using the emmeans package.

## 3 Results

### 3.1 Molecular Experiment

#### 3.1.1 HSP24

**FIGURE 3:**
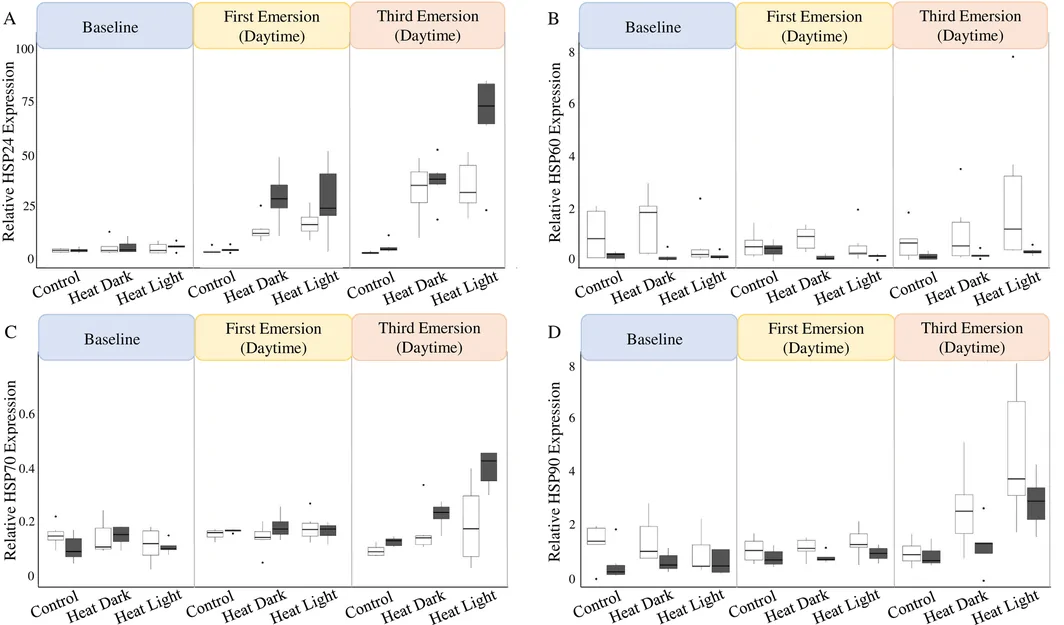
Effects of repeated heat exposure on *M. edulis* for three different treatments (control, heat dark and heat light), and two conditions (with endoliths in white, without endoliths in black) on the relative expression of (A) HSP24, (B) HSP60, (C) HSP70 and (D) HSP90 genes. Boxplots show the median, interquartile range, whiskers (1.5 × IQR) and outliers. Data are shown for *n* = 6 male mussels per treatment, condition and timepoint. Statistical analyses were performed using the Aligned Rank Transform (ART), followed by Holm‐adjusted post hoc pairwise comparisons.

Before heat exposure (baseline), HSP24 expression did not differ among treatments or conditions (Figure 3A; Table S3). After the first heat exposure (first emersion (daytime)), expression increased significantly in all heat‐stress treatments compared to control levels at that timepoint (with endoliths: heat dark *p* = 0.006, heat light *p* = 0.0012; without endoliths: heat dark *p* = 0.0001, heat light *p* = 0.0012) and baseline levels (with endoliths: heat light *p* = 0.02; without endoliths: heat dark *p* = 0.002, heat light *p* = 0.001). After the third heat exposure (third emersion (daytime)), HSP24 remained elevated across all heat treatments compared to both baseline (with endoliths: heat dark *p* = 0.0003, heat light *p* = 0.0001; without endoliths: heat dark *p* = 0.001, heat light *p* = 0.0001) and control levels at that time point (with endoliths: heat dark and heat light *p* = 0.0001; without endoliths: heat dark *p* = 0.001, heat light *p* = 0.0001). In addition, mussels without endoliths under the heat light treatment showed a further increase in HSP24 expression relative to the first exposure (*p* = 0.03). At this timepoint, mussels without endoliths exhibited higher expression than those with endoliths in the heat light treatment (*p* = 0.038).

#### 3.1.2 HSP60

Across all treatments and timepoints, mussels with endoliths consistently showed higher HSP60 expression than mussels without endoliths (overall: *p* = 0.0001; Table S3). When split by timepoint, this pattern remained, with significantly higher expression in mussels with endoliths before heat exposure (baseline, *p* = 0.005), after the first exposure (first emersion (daytime), *p* = 0.01) and after the third exposure (third emersion (daytime), *p* = 0.004). When comparing within each treatment (Figure 3B), HSP60 expression was higher in mussels with endoliths compared to those without endoliths in the heat dark treatment (*p* = 0.04). Over time, mussels with endoliths in the heat stress light treatment showed increased HSP60 expression, with levels after the third emersion significantly higher than before heat exposure (baseline, *p* = 0.04).

#### 3.1.3 HSP70

Baseline levels, as well as after the first emersion, no differences in HSP70 expression were detected among treatments or conditions (Figure 3C; Table S3). After the third emersion, mussels without endoliths in the heat light treatment showed significantly higher expression compared to their corresponding controls (*p* = 0.003) and compared to mussels with endoliths under the same treatment (*p* = 0.02). HSP70 expression in mussels without endoliths was also significantly higher compared to their baseline level (*p* = 0.008). Overall, mussels without endoliths showed higher HSP70 expression than mussels with endoliths after the third emersion (*p* = 0.003).

#### 3.1.4 HSP90

Across all treatments and timepoints, mussels with endoliths showed consistently higher HSP90 expression than mussels without endoliths (overall: *p* = 0.0008; Figure 3D; Table S3). When considering timepoints separately, this difference remained significant before heat exposure (baseline, *p* = 0.01) and after the first emersion (*p* = 0.01), but was no longer detected after the third emersion. Baseline expression and expression after the first emersion did not differ between treatments and conditions. After the third emersion, HSP90 expression increased under both heat treatments. Mussels with endoliths in the heat dark treatment expressed significantly higher levels than controls (*p* = 0.03). Mussels with and without endoliths in the heat light treatment also increased their HSP90 expression levels compared to their respective controls (with endoliths *p* = 0.0003; without endoliths *p* = 0.003), as well as compared to the first emersion (with endoliths *p* = 0.02; without endoliths *p* = 0.007).

### 3.2 Behavioural Experiment

#### 3.2.1 Gross Distance

**FIGURE 4:**
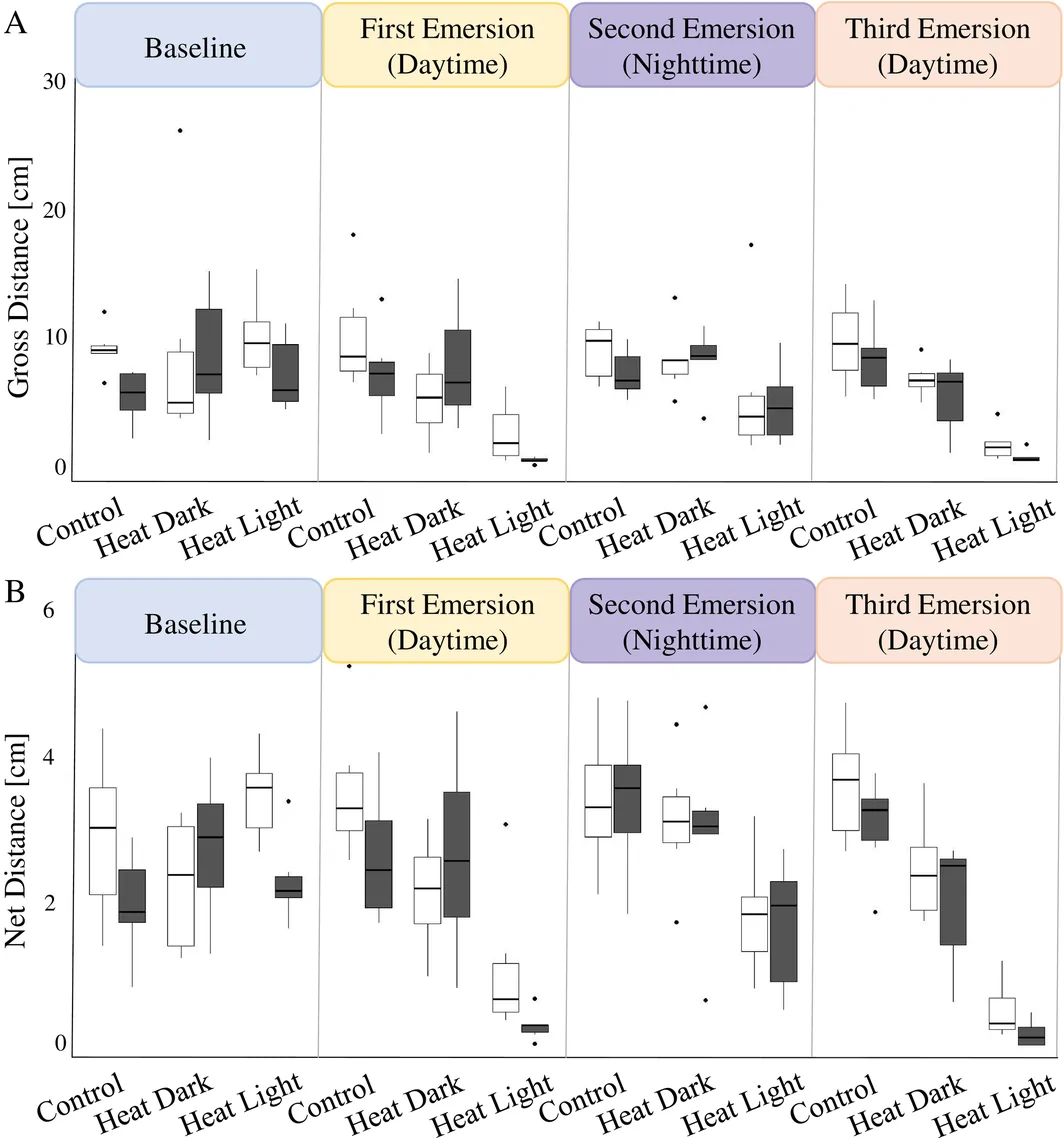
Effects of repeated heat exposure on *M. edulis* for three different treatments (control, heat dark and heat light), and two conditions (with endoliths in white, without endoliths in black) on (A) gross distance and (B) net distance crawled. Boxplots show the median, interquartile range, whiskers (1.5 × IQR) and outliers. Data are shown for *n* = 6 replicated behavioural arenas per treatment, condition and timepoint. Statistical analyses were performed using generalised linear mixed‐effects models, with arena included as a random effect to account for repeated measures over time, followed by pairwise comparisons using estimated marginal means with Tukey adjustment.

No significant differences in gross distance crawled were observed before heat exposure, neither among treatment nor between the two conditions (baseline, with and without endoliths; Figure 4A; Table S4). After the first emersion, mussels without endoliths in the heat light treatment exhibited significantly reduced movement compared with the same group before heat exposure (*p* = 0.013). Mussels without endoliths in this treatment recovered after the lower thermal stress during the second emersion (nighttime), with no significant differences among treatments or between conditions. Following the third heat exposure, movement under heat light decreased significantly (with endoliths *p* = 0.0001; without endoliths *p* = 0.008) relative to their respective baseline values. In addition, after the third emersion (daytime), both conditions under the heat light treatment moved significantly less than their corresponding controls (with endoliths *p* = 0.005; without endoliths *p* = 0.008).

#### 3.2.2 Net Distance

No significant differences in net distance crawled were observed before heat exposure (baseline), neither among treatment groups nor between the two conditions (with and without endoliths; Figure 4B; Table S4). After the first emersion, mussels under the heat light treatment exhibited significantly reduced movement compared to baseline levels of the same treatment and condition (with endoliths *p* = 0.0001; without endoliths *p* = 0.0005), and compared to their respective controls at that time (with endoliths *p* = 0.0001; without endoliths *p* = 0.0002). After the second emersion (nighttime), net distance remained significantly lower in both conditions under heat light compared with controls (with endoliths *p* = 0.02; without endoliths *p* = 0.002). After the third emersion, net distance decreased further under the heat light treatment relative to baseline levels and corresponding controls (with and without endoliths both *p* = 0.0001). In addition, mussels without endoliths showed a further decrease in movement compared to the second emersion (*p* = 0.02).

#### 3.2.3 Attachment Strength

**FIGURE 5:**
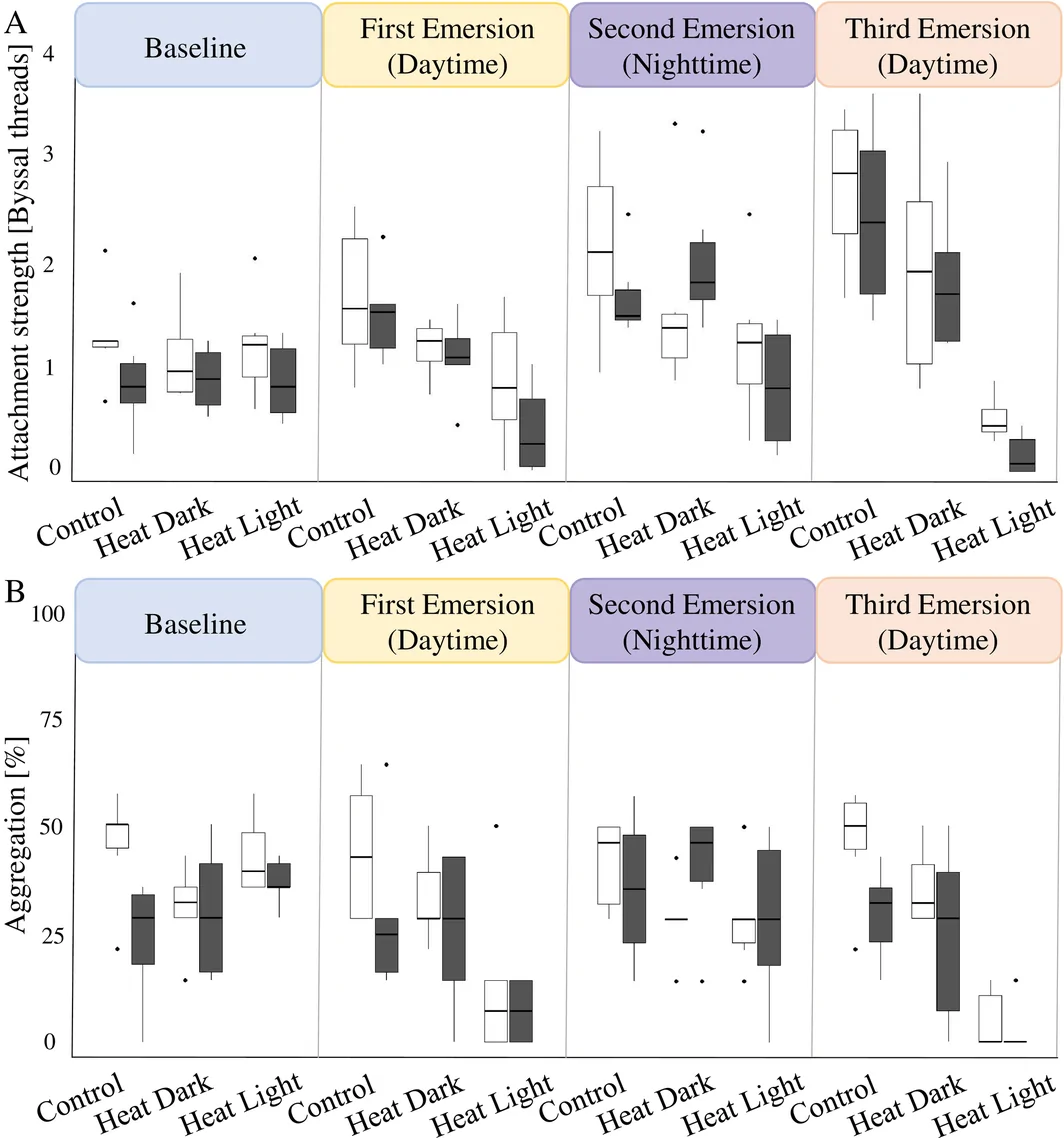
Effects of repeated heat exposure on *M. edulis* for three different treatments (control, heat dark and heat light), and two conditions (with endoliths in white, without endoliths in black) on (A) attachment strength and (B) percentage of mussels aggregated. Boxplots show the median, interquartile range, whiskers (1.5 × IQR) and outliers. Data are shown for *n* = 6 replicated behavioural arenas per treatment, condition and timepoint. Statistical analyses were performed using generalised linear mixed‐effects models, with arena included as a random effect to account for repeated measures over time, followed by pairwise comparisons using estimated marginal means with Tukey adjustment.

No significant differences in the number of byssal threads—as a proxy of attachment strength—were observed before heat exposure, neither among treatment groups, nor between the two conditions (baseline, with and without endoliths; Figure 5A; Table S4). After the first emersion (daytime), mussels without endoliths under the heat light treatment significantly reduced their byssal thread production compared to the control (*p* = 0.003). After the second emersion (nighttime), mussels without endoliths under the heat light treatment showed a significantly lower attachment compared to mussels without endoliths under the heat dark treatment (*p* = 0.0003). These mussels without endoliths under heat dark showed increased attachment relative to their baseline level (*p* = 0.0001) and compared to the same treatment after the first emersion (*p* = 0.015). After the third emersion (daytime), mussels under the heat light treatment showed a significantly lower attachment compared to their respective controls (with and without endoliths *p* = 0.0001) and to the respective heat dark treatments (with and without endoliths *p* = 0.0001). Over time, attachment strength increased in the controls: after the third emersion mussels showed a significantly higher attachment compared to the number of byssal threads at the baseline timepoint (with and without endoliths *p* = 0.0001). Similarly, mussels without endoliths under heat dark showed increased attachment compared to their baseline levels (*p* = 0.009).

#### 3.2.4 Aggregation

No significant differences in aggregation (% of mussels in aggregates) were observed before heat exposure, neither among treatment groups, nor between the two conditions (baseline, with and without endoliths) (Figure 5B; Table S4). After the first emersion, mussels under the heat light treatment exhibited a significantly reduced aggregation compared to their baseline levels (with endoliths *p* = 0.004; without endoliths *p* = 0.004). Groups with endoliths also showed a significant decrease compared to their respective control at that time (*p* = 0.004). After the second emersion (nighttime), aggregation showed no significant differences among treatments and conditions. However, after the third emersion (daytime), mussels without endoliths showed a significantly lower aggregation under the heat light treatment compared to the same group after the second emersion (*p* = 0.03). After the third emersion, aggregation decreased further under the heat light treatment relative to baseline levels and controls (with endoliths both *p* = 0.0001; without endoliths: baseline *p* = 0.0002, controls *p* = 0.03). In addition, mussels with endoliths in the heat light treatment showed a further decrease in aggregation compared to mussels with endoliths in the heat dark treatment (*p* = 0.004).

### 3.3 Survival Experiment

**FIGURE 6:**
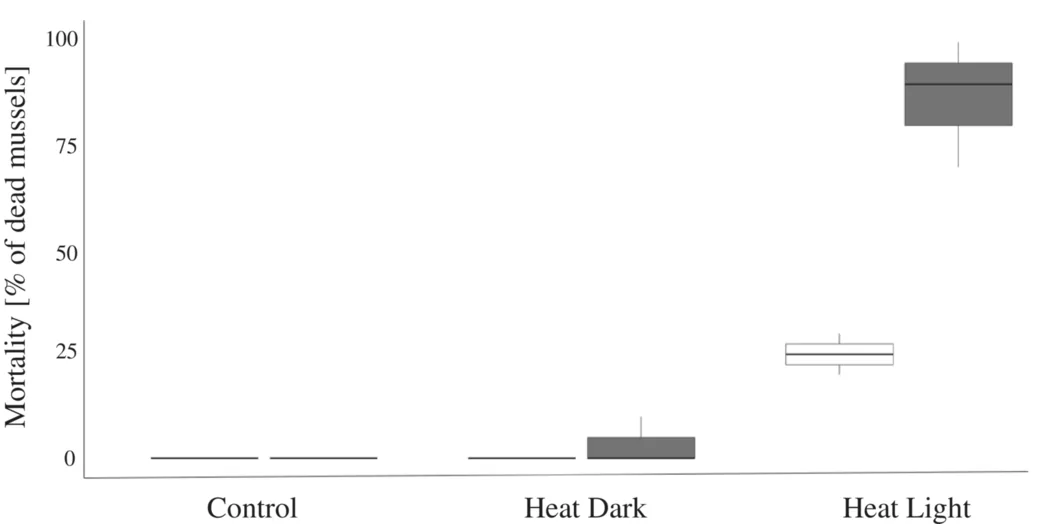
Effects of repeated heat exposure on *M. edulis* survival for three different treatments (control, heat dark and heat light), with and without endoliths (corroded in white; non‐corroded in black). Boxplots show the median, interquartile range and whiskers (1.5 × IQR). Data are shown for *n* = 20 mussels per treatment and condition. Statistical analyses were performed using a generalised linear model, followed by pairwise comparisons using estimated marginal means.

Significantly higher mortality was observed in mussels without endoliths under the heat light treatment compared to mussels with endoliths (*p* = 0.0001, Figure 6; Table S5). Mussels without endoliths under heat light further showed higher mortality than mussels without endoliths in both the heat dark and control treatments (both *p* = 0.0001). Similarly, mussels with endoliths in the heat light treatment exhibited higher mortality than those in the respective control (*p* = 0.009) and heat dark treatment (*p* = 0.009).

## 4 Discussion

Symbiotic interactions are increasingly recognised as critical modulators of animal responses to environmental stress, yet their role in shaping physiological and behavioural resilience remains poorly understood. Intertidal mussels such as *M. edulis* regularly face prolonged aerial exposure during low tide, often coinciding with stressful high temperatures (Denny et al. 2011; Helmuth 1998) that demand both behavioural and molecular strategies to withstand repeated thermal challenges (Anestis et al. 2007; Ioannou et al. 2009; Ng et al. 2021; Nicastro et al. 2012; Virgin et al. 2025). Endolithic symbioses are common in these mussels and can enhance host survival during thermal stress by whitening the shell surface and reducing heat absorption (Zardi et al. 2016, 2024). However, the molecular and behavioural mechanisms underlying this benefit remain unclear. Here, we extend previous findings on enhanced thermal survival by showing that endolithic microbial partners are also associated with altered molecular and behavioural responses in their hosts during repeated heat stress. Mussels harbouring endoliths exhibited distinct expression patterns of heat shock proteins (HSPs) compared to conspecifics without endoliths, and showed reduced behavioural impairments under repeated thermal stress. Together, these results suggest that the protective effects of endoliths extend beyond physical shell modifications, providing integrated physiological and behavioural buffering that may confer a selective advantage under fluctuating thermal conditions and future climate warming. However, this advantage likely depends on the present dynamics of the host‐symbiont interaction and should be viewed as context‐dependent, as such advantages may reflect transient outcomes of symbiont‐presence (i.e., immune elicitation) rather than a stable, evolved protective mechanism. Moreover, mussels with endoliths also exhibit consistent differences in shell condition, which naturally co‐occurs with microbial colonisation. As a result, the observed responses may reflect a combination of non‐exclusive mechanisms, including direct effects of endolithic microbial activity, indirect effects mediated by shell corrosion and associated changes in shell optical and structural properties. These mechanisms are not experimentally separable in the present study and should therefore be interpreted as alternative or interacting contributors rather than distinct causal pathways. What is however interesting here is that the inclusion of the heat‐dark treatment provides a first step towards disentangling these effects by maintaining thermal stress while removing solar irradiance, thereby reducing the contribution of shell‐mediated differences in heat absorption.

Because HSPs differ in their induction dynamics and stress specificity, they provide valuable insights into different types of stress. By examining HSP24, HSP60, HSP70 and HSP90, we were able to capture the functional diversity of the stress response. While HSP24 and HSP70 are generally regarded as heat‐specific markers (Feidantsis et al. 2020; Lockwood et al. 2010; Xu et al. 2014, 2022), HSP60 and HSP90 are involved in a broader spectrum of stressors, including thermal stress, metal exposure, elevated salinity or bacterial challenge (Bolhassani and Agi 2019; Ding et al. 2018; Kaufmann et al. 1991; Kim et al. 2009; Liu et al. 2019; Liu et al. 2016; Shi et al. 2016; Zhou et al. 2010). HSP60 has also been shown to be upregulated following shell damage (Sleight et al. 2018) and under bacterial challenge (Bolhassani and Agi 2019; Kaufmann et al. 1991; Liu et al. 2019; Zhou et al. 2010), making it a relevant marker for assessing stress potentially induced by endoliths.

Indeed, our results indicate an elevated expression of HSP60 and HSP90 in all individuals hosting endolithic microbes, both under control and thermal stress conditions. This constitutive upregulation suggests that mussels with endoliths experience a higher baseline level of stress, although whether it is triggered directly by endolithic microbes or indirectly through the secondary stress of shell corrosion remains unclear. Nevertheless, maintaining such elevated expression likely represents a sustained energetic investment, which may function as a preparative defence or frontloading mechanism that enhances preparedness for subsequent stressors (Barman et al. 2023; Dong et al. 2008; Greve et al. 2026; Hori and Fujishima 2003; Rinehart et al. 2006; Selbach et al. 2020). Such transcriptional frontloading may explain the contrasting expression patterns of HSP24 and HSP70 observed between individuals with and without endoliths during thermal stress. While HSP60 and HSP90 remained consistently elevated in mussels with endoliths, HSP24 and HSP70 responses were primarily driven by temperature. Importantly, their induction was reduced compared to mussels without endoliths. This pattern is consistent with transcriptional frontloading, where constitutively high expression of protective genes reduces the need for strong inducible stress responses during subsequent stressors (Collins et al. 2021). A recent host–parasite study in *M. edulis* showed moderate trematode infections and elevated temperature had additive negative effects on survival, while high parasite loads unexpectedly offset the detrimental effects of thermal stress (Selbach et al. 2020). This effect has been linked to parasite‐induced biomarker expression, potentially priming mussels to better withstand sudden heat shocks during warm low‐tide conditions. Similar patterns of parasite‐associated thermal tolerance have been reported and often attributed to stress priming or frontloading mechanisms (Barman et al. 2023; Greve et al. 2026; Hori and Fujishima 2003; Selbach et al. 2020). In this context, our elevated baseline HSP expression should not necessarily be viewed as evidence of improved condition, but rather as a potentially costly physiological state that may enhance resilience only when environmental stress occurs. Such context‐dependent trade‐offs are characteristic of stress priming and transcriptional frontloading, where prior investment in protection reduces the need for strong inducible stress responses during subsequent challenges (Barman et al. 2023; Dong et al. 2008; Hori and Fujishima 2003; Rinehart et al. 2006). Our findings provide support for this concept, suggesting that endoliths may similarly modulate host stress physiology in ways that enhance thermal resilience.

Differences in thermal response are likely reinforced by variation in heat load. Shell whitening induced by endolithic corrosion reduces solar absorption and thereby body temperature (Zardi et al. 2016). Importantly, the heat‐dark treatment allowed us to disentangle the contributions of higher baseline HSP60 and HSP90 expression and the albedo effect by maintaining heat stress while removing solar irradiance and thus the protective, light‐driven whitening effect. HSP24 and HSP70 levels in mussels with endoliths remained unchanged between heat‐light and heat‐dark conditions, contrary to mussels without endoliths, indicating that shell whitening substantially buffers body temperature by reducing solar heating. Field observations have shown that mussel body temperature can differ markedly from ambient air temperature (Helmuth et al. 2016; Lathlean et al. 2016) and is strongly influenced by solar radiation and microhabitat conditions, with shaded individuals maintaining temperatures closer to the surrounding air (Helmuth et al. 2010; Potter et al. 2013). This supports the rationale behind our heat‐dark treatment as a way to isolate the effect of solar‐driven shell heating. In line with this, mussels without endoliths exposed to heat in the dark showed HSP24 and HSP70 expression levels comparable to those of individuals with endoliths, reinforcing that shell whitening plays a key role in reducing thermal stress. Taken together, these mechanisms reveal a dual protective strategy—constitutive molecular buffering and thermal mitigation via the albedo effect—that synergistically enhances thermal tolerance in mussels with endoliths. Importantly, these different HSP expression patterns reflect distinct and non‐exclusive components of the stress response rather than a single unified mechanism. As such, reduced induction of HSP24 and HSP70 in mussels with endoliths, under heat stress, should not be interpreted in isolation as direct evidence of thermal buffering, but rather as part of a broader shift in stress physiology that includes both baseline energetic investment and reduced effective heat load under solar exposure.

Beyond its chaperone function, HSP70 is known to play a key role in innate immunity (Junprung et al. 2024; Zhang and Qi 2025). The absence of HSP70 upregulation in relation to endolithic presence therefore suggests that endoliths do not elicit a classical HSP70‐mediated immune response. This is unlikely to reflect a complete lack of host‐symbiont induced interaction, given the consistent upregulation of HSP60 and HSP90 in all individuals hosting endoliths. One possible explanation is that HSP70 involvement in immune activation may be constrained by counter‐selective pressures, as proposed for molluscs, where HSP70 induction levels appear to be shaped by trade‐offs among multiple physiological demands and divergent selection, acting across genotype or environmental conditions (Brokordt et al. 2015). Alternatively, HSP70 immune responses may be tissue‐specific and thus not fully captured here in mantle tissue.

The patterns of HSP expression were consistent with our behavioural observations, both indicating that mussels hosting endoliths experience lower levels of thermal stress than conspecifics without symbionts. Mussels with endoliths exhibited less pronounced behavioural impairments under repeated heat exposure, supporting the idea that these microbial associations mitigate sublethal stress responses. In intertidal organisms—including highly sedentary or sessile species—even subtle behavioural adjustments can confer substantial adaptive value (Zardi et al. 2015). Such responses allow individuals to avoid thermal extremes, optimise microhabitat use and modulate physiological stress, and are increasingly recognised as key contributors to ecological performance and long‐term survival (Tomanek 2002). Together, our findings suggest that endoliths provide integrated physiological and behavioural buffering, potentially conferring a selective advantage under fluctuating thermal conditions and future climate warming.

When challenged with adverse environmental conditions, mussels can actively relocate and form aggregates (Nicastro et al. 2008), a critical behavioural strategy that enhances survival by providing access to cooler, more stable microhabitats (Lathlean et al. 2016; Nicastro et al. 2012). In this context, mobility plays a central role in coping with both thermal and hydrodynamic stress in the intertidal zone. The ecological and evolutionary significance of mobility is reinforced by evidence that even small behavioural shifts can yield large benefits. In intertidal habitats, fine‐scale thermal heterogeneity is enormous and microhabitat variation over just a few centimetres can exceed latitudinal differences, giving mobile individuals access to microrefugia that significantly buffer thermal extremes (Denny et al. 2011; Helmuth 2002). Thus, mobility provides a front‐line defence against fluctuating and extreme changes, enhancing both survival and ecological performance in challenging intertidal environments. Here, we used net distance to quantify displacement from an individual's origin, regardless of the path taken and gross distance to quantify the total distance travelled to reflect the actual energetic effort of movement. Both metrics decreased significantly under heat‐light stress. Notably, mussels with endoliths maintained gross movement until the third heat exposure, whereas mussels without endoliths showed impaired movement after the first. This suggests that mussels hosting endoliths maintain normal activity and functional mobility longer under repeated heat stress, indicating higher resistance compared to conspecifics without endoliths. Such prolonged behavioural capacity may help preserve key defences against multiple environmental stressors. However, after several consecutive heat exposures, even endolith‐bearing mussels exhibited reduced movement, suggesting that a stress threshold is eventually reached.

Byssal thread production showed a response consistent with that observed for movement behaviour. Under heat‐light stress, non‐corroded mussels produced significantly fewer threads than controls, whereas mussels with endoliths showed no significant difference from their controls. Like aggregation and movement, sustained byssal attachment is an adaptive trait that enhances survival by providing mechanical stability against dislodgement (Nicastro et al. 2010) and maintaining access to favourable microhabitats within mussel beds (Carrington 2002; Zardi et al. 2007). Reduced attachment strength can increase vulnerability to both biotic stressors (e.g., predation) and abiotic stressors (e.g., wave action), ultimately compromising individual survival and population persistence (Norberg and Tedengren 1995; Waite 1983; Zardi et al. 2007). These findings suggest broader ecological implications: mussels with endoliths may be better able to maintain their position within mussel beds during increasingly frequent heat stress events, thereby contributing to population stability and resilience in intertidal communities (Bogdanski et al. 2024). While previous studies reported reduced attachment under thermal stress (Archambault et al. 2013; Li et al. 2020; Newcomb et al. 2019), our results showed a different pattern: heat‐stressed mussels exhibited only a slight decline in attachment over time, whereas controls displayed a significant increase across the three consecutive exposures. The increase in attachment among controls likely reflects a response to repeated handling. Individuals were removed from the arenas after each high‐tide simulation for low‐tide treatments, a procedure that mimicked hydrodynamic disturbance and is known to stimulate byssal thread production (Carrington 2002; Zardi et al. 2007).

The percentage of aggregated mussels declined with repeated heat and light exposure, with no direct differences between individuals with and without endoliths. Mussels typically aggregate to benefit from collective buffering, which generates more favourable thermal microclimates while also providing mechanical protection from wave action and other physical stressors (Helmuth 1998; Nicastro et al. 2012). The persistence of such structures relies on both resistance—through secure attachment that prevents disintegration—and resilience, as mobile individuals can move into empty spaces and restore aggregates, for instance after the death or dislodgement of conspecifics during storms (Nicastro et al. 2008). Although endoliths did not directly influence aggregation, the reduced mobility and weaker attachment observed under thermal stress in mussels without endoliths may ultimately constrain their ability to form and maintain aggregates, suggesting that this aggregation behaviour could be indirectly impaired under prolonged thermal stress.

Taken together, our behavioural results suggest that prolonged heat stress impairs the mussels' ability to move, attach and maintain aggregations. The presence of endolithic communities, however, may buffer these effects, helping to maintain adaptive responses and potentially contributing to the stability and resilience of mussel beds under increasingly stressful intertidal conditions. Importantly, under baseline non‐stressful conditions, we did not detect significant differences in any of the measured behavioural markers between mussels with and without endoliths. This suggests that endoliths do not impose detectable behavioural costs under normal conditions, but instead may confer benefits under thermal stress. These observations are consistent with previous literature, reporting no differences in aggregation and movement between mussels with and without endoliths under normal conditions (Bollina et al. 2026), while survival was higher in mussels with endoliths compared to those without endoliths under heat stress (Zardi et al. 2009). Overall, these findings indicate that endoliths may enhance functional resilience under thermal stress, without negatively affecting behaviour under baseline conditions.

Although elevated HSP expression and altered behavioural traits influence mussel performance and ecosystem functioning, the ultimate determinant of population persistence is survival. Consistent with our hypothesis and earlier studies (Gehman and Harley 2019; Lourenço et al. 2017; Monsinjon et al. 2021; Zardi et al. 2016, 2021), mussels with endolithic symbionts exhibited significantly higher survival under thermal stress. Four successive heat events at 30°C caused ~80% mortality in mussels without endoliths, compared to only ~25% in those hosting endoliths. Previous work has shown that repeated heat stress can decrease the thermal tolerance and drive high mortality (Feidantsis et al. 2020; Jones et al. 2009; Seuront et al. 2019). Our results demonstrate that mussels with endoliths are less susceptible to this cumulative stress, maintaining much higher survival across repeated exposures. Given that the severity and frequency of heat events is expected to intensify (Meehl and Tebaldi 2004; Rahmstorf and Coumou 2011; Stillman et al. 2025; Whalen et al. 2023), this enhanced survival and resilience represents a critical selective advantage.

This enhanced survival has important implications beyond individual hosts, particularly given the role of mussels as key ecosystem engineers (Arribas et al. 2014; Palomo et al. 2007). The persistence of mussel populations therefore underpins the resilience of entire intertidal communities. However, under future climate scenarios, where thermal stress events are projected to become increasingly frequent and severe (Meehl and Tebaldi 2004; Rahmstorf and Coumou 2011), intertidal mussels are expected to face heightened mortality (Petes et al. 2007; Tsuchiya 1983; Wagner et al. 2024). By enhancing thermal tolerance and improving the capacity of mussels to withstand extreme conditions, endoliths may play a pivotal role not only in shaping long‐term ecological and evolutionary dynamics of mussel populations, but also in sustaining the diverse microhabitats and ecosystem services supported by mussel beds. Recent evidence further suggests that the cooling effects of endolithic shell whitening can extend to infauna residing within mussel beds (Dievart et al. 2022; Zardi et al. 2023), indicating that the benefits of this association propagate beyond the host itself. Although regional biogeographic context often plays a stronger role in structuring these associated macrofaunal communities, the ability of endoliths to influence both host physiology and the microclimate within mussel beds highlights their potential to modulate the engineering capacity of mussel populations, with cascading effects for biodiversity and ecosystem functioning under climate change (Dievart et al. 2025).

Our study emphasises the value of a multidisciplinary approach that integrates molecular, behavioural and survival responses to capture the full scope of how symbiotic associations shape organismal resilience. While molecular markers such as HSPs reveal the hidden physiological costs and preparative defences triggered by endolithic communities, behavioural assays highlight functional consequences for mobility, attachment and aggregation, and survival experiments provide the ultimate measure of fitness under thermal stress. Together, these complementary perspectives show that the benefits of endoliths extend well beyond shell whitening, embedding protection at multiple levels of biological organisation.

Importantly, the implications are not limited to mussels alone as endoliths are a widespread phenomenon across calcifying taxa (Dievart et al. 2022; Golubic et al. 1975). This underscores the potential for similar host‐symbiont interactions to play underappreciated roles in modulating resilience in other systems.

## Author Contributions

Sarah Bollina, Katy Nicastro, Gerardo Zardi and Virginie Cuvillier conceived the ideas and designed the methodology; Sarah Bollina, Gerardo Zardi, Katy Nicastro, Virginie Cuvillier, Chloé Tilliette, Emma Brigant and Anne‐Catherine Holl collected the data; Sarah Bollina curated the data and performed the formal analyses; Katy Nicastro and Virginie Cuvillier acquired funding; Katy Nicastro and Virginie Cuvillier provided resources; Katy Nicastro, Gerardo Zardi and Virginie Cuvillier supervised the research; Sarah Bollina prepared the visualisations and led the writing of the manuscript. All authors contributed critically to the drafts and gave final approval for publication.

## Funding

This research was funded by FEDER project HDF009171 ECOMIC and further supported by ANR (project SAN22202).

## Ethics Statement

Specimens of *Mytilus edulis* were collected within their respective natural habitats. *M. edulis* is an invertebrate species that is not covered by Directive 2010/63/EU of the European Parliament and of the Council on the protection of animals used for scientific purposes. Therefore, no institutional or national ethical approval was required for the collection or use of these animals in this study. The ASAB/ABS guidelines for ethical treatment of nonhuman animals used in behavioural studies were followed throughout the experiment.

## Conflicts of Interest

The authors declare no conflicts of interest.

## Supporting information


**Table S1:** Temperature increase during low‐tide simulation for 30°C light and dark treatments.
**Table S2:** Primer sequences for HSP analyses.
**Table S3:** Pairwise post hoc comparison results for HSP24, HS60, HSP70 and HSP90.
**Table S4:** Pairwise post hoc comparison results for gross and net distance, attachment strength and aggregation.
**Table S5:** Pairwise post hoc comparison results for survival.

## Data Availability

The datasets supporting the conclusions of this article are provided on Zenodo (https://doi.org/10.5281/zenodo.22008865).
