# Supplementary material for: With a Little Help From Their Friends: Endolithic Microbial Communities Modulate Mussel Survival, Behaviour and Molecular Responses Under Thermal Stress
Source: Mol Ecol. 2026 Aug 21;35(16):e70524. doi: 10.1111/mec.70524 (PMC13498774; doi:10.1111/mec.70524)
Supplement: Supplementary file 1 [file MEC-35-e70524-s001.docx]

**Supplementary Material:**

*Table 1S. Temperature increase during low-tide simulation for 30°C light and dark treatments.*

| Start | 18°C |
| --- | --- |
| 0.5h | 21°C |
| 1h | 24.7°C |
| 1.5h | 26.8°C |
| 2h | 27.7°C |
| 2.5h | 28.5°C |
| 3h | 29.4°C |
| 3.5h | 30.2°C |
| 4h | 30.9°C |
| 4.5h | 31.6°C |
| 5h | 32.1°C |
| 5.5h | 32.7°C |
| 6h | 33.2°C |

*Table 2S. Primer sequences for HSP analyses.*

| **Gene** | **5’3’ sequence** | **Tm(°C)** | **ref** |
| --- | --- | --- | --- |
| HSP24 | ACGTTTGTCCCAGTGCTTTGGAA | 60 | Bultelle et al., 2021 |
|  | GGGTGACGACCATACCCATAACC |  |  |
| HSP60 | GGAGCAGATGCAAGAGCTATGATG | 60 | Bultelle et al., 2021 |
|  | GATTTGGCAACTGTAACTCCGTC |  |  |
| HSP70 | GGGTGGTGGAACTTTTGATG | 57 | Grimmelpont et al., 2024 |
|  | CTCTTTGCCCTTTCACAAGC |  |  |
| HSP90b | TCCCTGACCAATGATTGGGAGGA | 60 | Bultelle et al., 2021 |
|  | CATGTCAAATGGGGCTCTCTTTGG |  |  |
| Rps4  *ribosomal protein S4* | TGGGTTATCGAGGGCGTAG | 58 | Salatiello et al., 2022 |
|  | TCCCTTAGTTTGTGAGGACCTG |  |  |
| Rpl14  *ribosomal protein L14* | CAATGCCAAGATCCCTCCGTAC | 58 | Salatiello et al., 2022 |
|  | AGCTTGCTTGGCCTTCATCAG |  |  |

*Table 3S. Pairwise Post hoc comparison results for HSP24, HS60, HSP70, and HSP90.*

|  | | HSP24 | HSP60 | HSP70 | HSP90 |
| --- | --- | --- | --- | --- | --- |
| Baseline | Baseline Control with Endoliths vs Baseline Control without Endoliths | 1 | 0.91 | 0.49 | 0.29 |
|  | Baseline Heat Dark with Endoliths vs Baseline Heat Dark without Endoliths | 0.99 | **0.04** | 0.99 | 0.52 |
|  | Baseline Heat Light with Endoliths vs Baseline Heat Light without Endoliths | 0.87 | 0.87 | 0.99 | 0.98 |
|  | Baseline Control with Endoliths vs Baseline Heat Dark with Endoliths | 0.99 | 0.84 | 0.99 | 0.99 |
|  | Baseline Control with Endoliths vs Baseline Heat Light with Endoliths | 0.99 | 1 | 0.89 | 0.97 |
|  | Baseline Heat Dark with Endoliths vs Baseline Heat Light with Endoliths | 0.99 | 0.76 | 0.98 | 0.84 |
|  | Baseline Control without Endoliths vs Baseline Heat Dark withouth Endoliths | 0.97 | 0.91 | 0.49 | 0.95 |
|  | Baseline Control without Endoliths vs Baseline Heat Light without Endoliths | 0.83 | 0.99 | 0.99 | 0.98 |
|  | Baseline Heat Dark without Endoliths vs Baseline Heat Light without Endoliths | 0.99 | 0.98 | 0.74 | 1 |
| 1^st^ Emersion | 1st Emersion Control with Endoliths vs 1st Emersion Control without Endoliths | 1 | 0.99 | 0.93 | 0.71 |
|  | 1st Emersion Heat Dark with Endoliths vs 1st Emersion Heat Dark without Endoliths | 0.31 | 0.06 | 0.79 | 0.71 |
|  | 1st Emersion Heat Light with Endoliths vs 1st Emersion Heat Light without Endoliths | 1 | 0.55 | 1 | 0.75 |
|  | 1st Emersion Control with Endoliths vs 1st Emersion Heat Dark with Endoliths | **0.006** | 0.85 | 1 | 0.99 |
|  | 1st Emersion Control with Endoliths vs 1st Emersion Heat Light with Endoliths | **0.001** | 0.99 | 0.88 | 0.97 |
|  | 1st Emersion Heat Dark with Endoliths vs 1st Emersion Heat Light with Endoliths | 1 | 0.6 | 0.88 | 0.99 |
|  | 1st Emersion Control without Endoliths vs 1st Emersion Heat Dark withouth Endoliths | **0.0001** | 0.6 | 0.99 | 1 |
|  | 1st Emersion Control without Endoliths vs 1st Emersion Heat Light without Endoliths | **0.001** | 0.6 | 1 | 0.96 |
|  | 1st Emersion Heat Dark without Endoliths vs 1st Emersion Heat Light without Endoliths | 1 | 0.99 | 0.99 | 0.99 |
| 3^rd^ Emersion | 3rd Emersion Control with Endoliths vs 3rd Emersion Control without Endoliths | 0.28 | 0.32 | 0.51 | 0.99 |
|  | 3rd Emersion Heat Dark with Endoliths vs 3rd Emersion Heat Dark without Endoliths | 1 | 0.6 | 0.54 | 0.22 |
|  | 3rd Emersion Heat Light with Endoliths vs 3rd Emersion Heat Light without Endoliths | **0.038** | 0.44 | **0.02** | 0.89 |
|  | 3rd Emersion Control with Endoliths vs 3rd Emersion Heat Dark with Endoliths | **0.0001** | 1 | 0.15 | **0.03** |
|  | 3rd Emersion Control with Endoliths vs 3rd Emersion Heat Light with Endoliths | **0.0001** | 0.62 | 0.31 | **0.0003** |
|  | 3rd Emersion Heat Dark with Endoliths vs 3rd Emersion Heat Light with Endoliths | 1 | 0.71 | 1 | 0.43 |
|  | 3rd Emersion Control without Endoliths vs 3rd Emersion Heat Dark without Endoliths | **0.001** | 0.99 | 0.24 | 0.86 |
|  | 3rd Emersion Control without Endoliths vs 3rd Emersion Heat Light without Endoliths | **0.0001** | 0.48 | **0.003** | **0.003** |
|  | 3rd Emersion Heat Dark without Endoliths vs 3rd Emersion Heat Light without Endoliths | 0.09 | 0.85 | 0.32 | 0.06 |
| Across Timepoints | Baseline Control with Endoliths vs 1st Emersion Control with Endoliths | 0.95 | 0.99 | 0.71 | 0.99 |
|  | Baseline Control with Endoliths vs 3rd Emersion Control with Endoliths | 0.11 | 0.99 | 0.06 | 0.97 |
|  | Baseline Control without Endoliths vs 1st Emersion Control without Endoliths | 0.99 | 0.65 | 0.55 | 0.85 |
|  | Baseline Control without Endoliths vs 3rd Emersion Control without Endoliths | 0.96 | 0.99 | 0.84 | 0.63 |
|  | Baseline Heat Dark with Endoliths vs 1st Emersion Heat Dark with Endoliths | 0.28 | 1 | 1 | 1 |
|  | Baseline Heat Dark with Endoliths vs 3rd Emersion Heat Dark with Endoliths | **0.0003** | 1 | 1 | 1 |
|  | Baseline Heat Dark without Endoliths vs 1st Emersion Heat Dark without Endoliths | **0.002** | 1 | 1 | 1 |
|  | Baseline Heat Dark without Endoliths vs 3rd Emersion Heat Dark without Endoliths | **0.0001** | 0.94 | 0.64 | 0.54 |
|  | Baseline Heat Light with Endoliths vs 1st Emersion Heat Light with Endoliths | **0.02** | 1 | 1 | 0.51 |
|  | Baseline Heat Light with Endoliths vs 3rd Emersion Heat Light with Endoliths | **0.0001** | **0.04** | 1 | **0.0001** |
|  | Baseline Heat Light without Endoliths vs 1st Emersion Heat Light without Endoliths | **0.001** | 1 | 1 | 1 |
|  | Baseline Heat Light without Endoliths vs 3rd Emersion Heat Light without Endoliths | **0.0001** | 0.19 | **0.008** | **0.0001** |
|  | 1st Emersion Control with Endoliths vs 3rd Emersion Control with Endoliths | 0.48 | 1 | 0.6 | 0.99 |
|  | 1st Emersion Heat Dark with Endoliths vs 3rd Emersion Heat Dark with Endoliths | 0.17 | 1 | 1 | 1 |
|  | 1st Emersion Heat Light with Endoliths vs 3rd Emersion Heat Light with Endoliths | 0.31 | 0.76 | 1 | **0.02** |
|  | 1st Emersion Control without Endoliths vs 3rd Emersion Control without Endoliths | 0.99 | 0.87 | 0.99 | 0.99 |
|  | 1st Emersion Heat Dark without Endoliths vs 3rd Emersion Heat Dark without Endoliths | 1 | 1 | 0.69 | 1 |
|  | 1st Emersion Heat Light without Endoliths vs 3rd Emersion Heat Light without Endoliths | **0.03** | 0.68 | 0.09 | **0.007** |

*Table 4S. Pairwise Post hoc comparison results for Gross and Net Distance, Attachment Strength, and Aggregation.*

|  | | Gross Distance | Net Distance | Attachment | Aggregation |
| --- | --- | --- | --- | --- | --- |
| Baseline | Baseline Control with Endoliths vs Baseline Control without Endoliths | 0.96 | 0.95 | 0.99 | 0.3 |
|  | Baseline Heat Dark with Endoliths vs Baseline Heat Dark without Endoliths | 1 | 1 | 1 | 1 |
|  | Baseline Heat Light with Endoliths vs Baseline Heat Light without Endoliths | 0.97 | 0.68 | 1 | 1 |
|  | Baseline Control with Endoliths vs Baseline Heat Dark with Endoliths | 1 | 0.99 | 1 | 0.95 |
|  | Baseline Control with Endoliths vs Baseline Heat Light with Endoliths | 1 | 1 | 0.99 | 1 |
|  | Baseline Heat Dark with Endoliths vs Baseline Heat Light with Endoliths | 1 | 0.62 | 1 | 0.99 |
|  | Baseline Control without Endoliths vs Baseline Heat Dark withouth Endoliths | 0.99 | 0.99 | 1 | 1 |
|  | Baseline Control without Endoliths vs Baseline Heat Light without Endoliths | 1 | 1 | 1 | 0.98 |
|  | Baseline Heat Dark without Endoliths vs Baseline Heat Light without Endoliths | 1 | 1 | 1 | 1 |
| 1^st^ Emersion | 1st Emersion Control with Endoliths vs 1st Emersion Control without Endoliths | 0.99 | 0.96 | 1 | 1 |
|  | 1st Emersion Heat Dark with Endoliths vs 1st Emersion Heat Dark without Endoliths | 0.99 | 1 | 1 | 1 |
|  | 1st Emersion Heat Light with Endoliths vs 1st Emersion Heat Light without Endoliths | 1 | 0.99 | 0.96 | 1 |
|  | 1st Emersion Control with Endoliths vs 1st Emersion Heat Dark with Endoliths | 0.53 | 0.31 | 1 | 0.99 |
|  | 1st Emersion Control with Endoliths vs 1st Emersion Heat Light with Endoliths | **0.01** | **0.0001** | 0.43 | **0.004** |
|  | 1st Emersion Heat Dark with Endoliths vs 1st Emersion Heat Light with Endoliths | 0.99 | 0.86 | 0.98 | 0.42 |
|  | 1st Emersion Control without Endoliths vs 1st Emersion Heat Dark withouth Endoliths | 1 | 1 | 0.99 | 1 |
|  | 1st Emersion Control without Endoliths vs 1st Emersion Heat Light without Endoliths | **0.05** | **0.0002** | **0.003** | 0.3 |
|  | 1st Emersion Heat Dark without Endoliths vs 1st Emersion Heat Light without Endoliths | **0.017** | **0.0001** | 0.26 | 0.55 |
| 2^nd^ Emersion | 2nd Emersion Control with Endoliths vs 2nd Emersion Control without Endoliths | 1 | 1 | 0.99 | 1 |
|  | 2nd Emersion Heat Dark with Endoliths vs 2nd Emersion Heat Dark without Endoliths | 1 | 1 | 0.88 | 0.99 |
|  | 2nd Emersion Heat Light with Endoliths vs 2nd Emersion Heat Light without Endoliths | 1 | 1 | 0.99 | 1 |
|  | 2nd Emersion Control with Endoliths vs 2nd Emersion Heat Dark with Endoliths | 1 | 1 | 0.89 | 0.98 |
|  | 2nd Emersion Control with Endoliths vs 2nd Emersion Heat Light with Endoliths | 0.99 | 0.29 | 0.24 | 0.98 |
|  | 2nd Emersion Heat Dark with Endoliths vs 2nd Emersion Heat Light with Endoliths | 0.99 | 1 | 1 | 1 |
|  | 2nd Emersion Control without Endoliths vs 2nd Emersion Heat Dark withouth Endoliths | 1 | 1 | 0.99 | 1 |
|  | 2nd Emersion Control without Endoliths vs 2nd Emersion Heat Light without Endoliths | 1 | 0.15 | 0.17 | 1 |
|  | 2nd Emersion Heat Dark without Endoliths vs 2nd Emersion Heat Light without Endoliths | 0.98 | 0.64 | **0.0003** | 0.99 |
| 3^rd^ Emersion | 3rd Emersion Control with Endoliths vs 3rd Emersion Control without Endoliths | 1 | 1 | 0.99 | 0.8 |
|  | 3rd Emersion Heat Dark with Endoliths vs 3rd Emersion Heat Dark without Endoliths | 1 | 1 | 1 | 0.99 |
|  | 3rd Emersion Heat Light with Endoliths vs 3rd Emersion Heat Light without Endoliths | 1 | 1 | 0.99 | 1 |
|  | 3rd Emersion Control with Endoliths vs 3rd Emersion Heat Dark with Endoliths | 0.99 | 0.7 | 0.27 | 0.99 |
|  | 3rd Emersion Control with Endoliths vs 3rd Emersion Heat Light with Endoliths | **0.005** | **0.0001** | **0.0001** | **0.0001** |
|  | 3rd Emersion Heat Dark with Endoliths vs 3rd Emersion Heat Light with Endoliths | 0.52 | **0.009** | **0.0001** | **0.004** |
|  | 3rd Emersion Control without Endoliths vs 3rd Emersion Heat Dark withouth Endoliths | 0.99 | 0.86 | 0.95 | 1 |
|  | 3rd Emersion Control without Endoliths vs 3rd Emersion Heat Light without Endoliths | **0.008** | **0.0001** | **0.0001** | **0.027** |
|  | 3rd Emersion Heat Dark without Endoliths vs 3rd Emersion Heat Light without Endoliths | 0.59 | **0.0001** | **0.0001** | 0.21 |
| Across Timepoints | Baseline Control with Endoliths vs 1st Emersion Control with Endoliths | 1 | 0.99 | 1 | 1 |
|  | Baseline Control with Endoliths vs 2nd Emersion Control with Endoliths | 1 | 1 | 0.19 | 1 |
|  | Baseline Control with Endoliths vs 3rd Emersion Control with Endoliths | 1 | 0.99 | **0.0001** | 1 |
|  | Baseline Control without Endoliths vs 1st Emersion Control without Endoliths | 1 | 0.99 | 0.88 | 1 |
|  | Baseline Control without Endoliths vs 2nd Emersion Control without Endoliths | 1 | 0.21 | 0.37 | 0.99 |
|  | Baseline Control without Endoliths vs 3rd Emersion Control without Endoliths | 0.99 | 0.63 | **0.0001** | 1 |
|  | Baseline Heat Dark with Endoliths vs 1st Emersion Heat Dark with Endoliths | 0.83 | 1 | 1 | 1 |
|  | Baseline Heat Dark with Endoliths vs 2nd Emersion Heat Dark with Endoliths | 1 | 0.97 | 0.99 | 1 |
|  | Baseline Heat Dark with Endoliths vs 3rd Emersion Heat Dark with Endoliths | 0.99 | 1 | 0.11 | 1 |
|  | Baseline Heat Dark without Endoliths vs 1st Emersion Heat Dark without Endoliths | 1 | 1 | 1 | 1 |
|  | Baseline Heat Dark without Endoliths vs 2nd Emersion Heat Dark without Endoliths | 1 | 1 | **0.0001** | 0.99 |
|  | Baseline Heat Dark without Endoliths vs 3rd Emersion Heat Dark without Endoliths | 0.95 | 0.99 | **0.009** | 1 |
|  | Baseline Heat Light with Endoliths vs 1st Emersion Heat Light with Endoliths | **0.002** | **0.0001** | 1 | **0.004** |
|  | Baseline Heat Light with Endoliths vs 2nd Emersion Heat Light with Endoliths | 0.81 | 0.06 | 0.99 | 0.93 |
|  | Baseline Heat Light with Endoliths vs 3rd Emersion Heat Light with Endoliths | **0.0001** | **0.0001** | 0.97 | **0.0001** |
|  | Baseline Heat Light without Endoliths vs 1st Emersion Heat Light without Endoliths | **0.01** | **0.0005** | 0.72 | **0.004** |
|  | Baseline Heat Light without Endoliths vs 2nd Emersion Heat Light without Endoliths | 0.99 | 0.99 | 1 | 1 |
|  | Baseline Heat Light without Endoliths vs 3rd Emersion Heat Light without Endoliths | **0.008** | **0.0001** | 0.23 | **0.0002** |
|  | 1st Emersion Control with Endoliths vs 2nd Emersion Control with Endoliths | 1 | 1 | 0.91 | 1 |
|  | 1st Emersion Heat Dark with Endoliths vs 2nd Emersion Heat Dark with Endoliths | 0.97 | 0.89 | 1 | 1 |
|  | 1st Emersion Heat Light with Endoliths vs 2nd Emersion Heat Light with Endoliths | 0.97 | 0.99 | 0.98 | 0.86 |
|  | 1st Emersion Control without Endoliths vs 2nd Emersion Control without Endoliths | 1 | 0.99 | 1 | 1 |
|  | 1st Emersion Heat Dark without Endoliths vs 2nd Emersion Heat Dark without Endoliths | 1 | 1 | **0.015** | 0.93 |
|  | 1st Emersion Heat Light without Endoliths vs 2nd Emersion Heat Light without Endoliths | 0.64 | 0.18 | 0.95 | 0.24 |
|  | 1st Emersion Control with Endoliths vs 3rd Emersion Control with Endoliths | 1 | 1 | **0.0003** | 1 |
|  | 1st Emersion Heat Dark with Endoliths vs 3rd Emersion Heat Dark with Endoliths | 1 | 1 | 0.55 | 1 |
|  | 1st Emersion Heat Light with Endoliths vs 3rd Emersion Heat Light with Endoliths | 1 | 0.99 | 0.99 | 1 |
|  | 1st Emersion Control without Endoliths vs 3rd Emersion Control without Endoliths | 1 | 1 | 0.05 | 1 |
|  | 1st Emersion Heat Dark without Endoliths vs 3rd Emersion Heat Dark without Endoliths | 1 | 0.99 | 0.35 | 1 |
|  | 1st Emersion Heat Light without Endoliths vs 3rd Emersion Heat Light without Endoliths | 1 | 1 | 1 | 1 |
|  | 2nd Emersion Control with Endoliths vs 3rd Emersion Control with Endoliths | 1 | 1 | 0.45 | 1 |
|  | 2nd Emersion Heat Dark with Endoliths vs 3rd Emersion Heat Dark with Endoliths | 1 | 0.99 | 0.98 | 1 |
|  | 2nd Emersion Heat Light with Endoliths vs 3rd Emersion Heat Light with Endoliths | 0.6 | 0.33 | 0.22 | 0.09 |
|  | 2nd Emersion Control without Endoliths vs 3rd Emersion Control without Endoliths | 1 | 1 | 0.36 | 1 |
|  | 2nd Emersion Heat Dark without Endoliths vs 3rd Emersion Heat Dark without Endoliths | 0.96 | 0.86 | 1 | 0.86 |
|  | 2nd Emersion Heat Light without Endoliths vs 3rd Emersion Heat Light without Endoliths | 0.55 | 0.08 | 0.61 | **0.03** |

*Table 5S. Pairwise Post hoc comparison results for Survival.*

|  |  | Survival |
| --- | --- | --- |
| 4^th^ Emersion | Control with Endoliths vs Control without Endoliths | 1 |
|  | Heat Dark with Endoliths vs Heat Dark without Endoliths | 0.99 |
|  | Heat Light with Endoliths vs Heat Light without Endoliths | **0.0001** |
|  | Control with Endoliths vs Heat Dark with Endoliths | 1 |
|  | Control with Endoliths vs Heat Light with Endoliths | **0.009** |
|  | Heat Dark with Endoliths vs Heat Light with Endoliths | **0.009** |
|  | Control without Endoliths vs Heat Dark withouth Endoliths | 0.99 |
|  | Control without Endoliths vs Heat Light without Endoliths | **0.0001** |
|  | Heat Dark without Endoliths vs Heat Light without Endoliths | **0.0001** |
